# Fabricating Paper Based Devices Using Correction Pens

**DOI:** 10.1038/s41598-018-38308-6

**Published:** 2019-02-11

**Authors:** Naresh Kumar Mani, Anusha Prabhu, Sujay Kumar Biswas, Suman Chakraborty

**Affiliations:** 1 Department of Biotechnology, Manipal Institute of Technology, Manipal Academy of Higher Education, Manipal, 576104 Karnataka India; 2 School of Medical Science and Technology, Indian Institute of Technology Kharagpur, Kharagpur, 721302 India; 3 Department of Mechanical Engineering, Indian Institute of Technology Kharagpur, Kharagpur, 721302 India

## Abstract

We present a rapid (<10 s), cost-effective, unique single-step method for fabricating paper-based devices without necessitating any expensive instrumentation, simply by deploying correction pens that are otherwise commonly used for masking typos in printed or written matters. The marked regions formed by deposits from the correction pen demonstrate ubiquitous flow resistances to typical aqueous solutions and organic solvents in the transverse direction, resulting in a preferential bulk flow along the axial direction of the paper channels ‘fabricated’ in the process. Considering the simplicity and cost-effectiveness of this platform, it is deemed to be ideal for (bio) chemical sensing and point-of-care diagnostics in resource-limited settings.

## Introduction

Paper, as a versatile substrate, has garnered attention in the miniaturization and microfluidics community due to its low cost, passive fluid transport via capillary action, porosity, biodegradability, biocompatibility and random network structure. The pioneering efforts of Whiteside’s group^1–3^ have transformed a simple paper into paper-based analytical devices in association with fluorescence, colorimetric, and electrochemical detection systems^4,5^. Afterwards, many other interesting phenomena like electrically modulated flow control^6^, micromixing^7,8^, separation^9^, energy generation^10–12^ etc. have been explored and studied using paper-based device. The contribution of this device is significant in various fields including clinical, food industries, smart phone based sensing, environmental applications and point-of-care tests^13–15^. Of late, scientists and researchers have found a deep interest in studying the fundamentals of fluid transport through the random porous structure of the paper matrix^16–18^.

The hydrophilic nature of the paper pores, coupled with patterning of hydrophobic barriers across the direction of preferential transport, is a quintessential consideration in constructing paper-based analytical devices. Plethora of expensive techniques has been used for fabricating hydrophobic barriers on paper, including photolithography^1,19,20^, flexography printing^21^, plasma treatment^22–24^, cutting^25,26^ and vapour phase deposition^27–29^. Various low-cost techniques like PDMS printing^30^, wax printing^31,32^, ink-jet printing^33–36^, wax dipping^37,38^, screen printing^39,40^ and stamping^41,42^ have also been developed. The common drawbacks with the aforementioned techniques are high cost, long fabrication time, expertise, requirement of external armamentarium like laser, oven, printer and stamps. Access to such instruments still remains a challenge in resource-limited laboratories, and consequently, this may end up in delayed diagnosis or detection of ailments using body-fluid based procedures^43,44^. One way to overcome this problem is by free-hand drawing, where a novice can fabricate paper-based devices^45,46^.

In this work, we describe a rapid, frugal and facile proof-of-concept of fabricating paper-based analytical devices using correction pen. Traditionally, correction pen is used as an agent to mask errors in printed or written text. Exact composition of correction pen ink is a trade secret. However, literature suggests that it is a mixture of titanium dioxide, solvents, resins and colorant^47^. To the best of our knowledge, this combination has never been leveraged as a barrier patterning agent on paper. Our one-step fabrication involves direct manual deposition (i.e, free-hand drawing) of the correction fluid on filter paper. Strikingly, this method requires no heating, no complex instrumentation, and no trained personnel.

## Results and Discussions

### Fabrication of Correction pen-based barrier on paper

**Figure 1:**
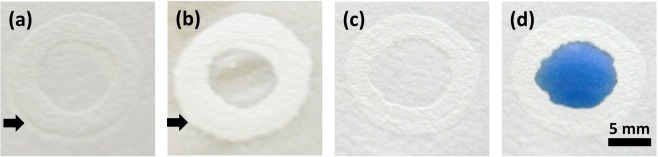
One-step fabrication of paper-based devices. (**a**) Barrier depicted by an arrow (**b**) wetted with water (**c**) without dye (**d**) with dye.

**Figure 2:**
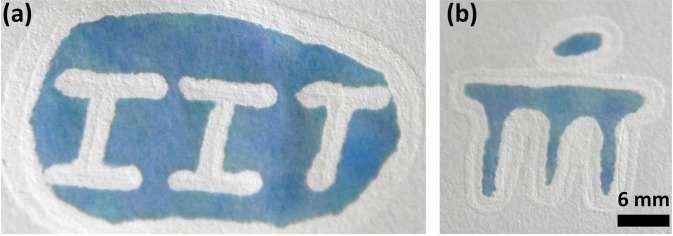
Demonstration- printing the Institutional acronyms of the contributing authors.

First, we have constructed circular devices through direct patterning of correction liquid from the pen on filter paper. After solvent evaporation (~15 min), we observed that Titanium dioxide gets embedded on paper and blocks the perforated pores, consequently forming a visible hydrophobic barrier (Fig. 1a). This has been confirmed by wetting the device with water (Fig. 1b). When coloured water was introduced to the fabricated device, the hydrophobic barriers have confined the same without any disruption or leakage (Fig. 1c,d). Through free hand-drawing, we have designed our respective institute’s acronyms and further checked their water confinement ability (Fig. 2). We have also designed a channel and assessed the wicking ability of paper device (Supplementary Fig. 1). This method offers advantages like less fabrication time (10 s for circular device), one-time deposition of correction fluid, 1 mm resolution and no dependence of heating step.

### Barrier’s chemical compatibility

**Figure 3:**
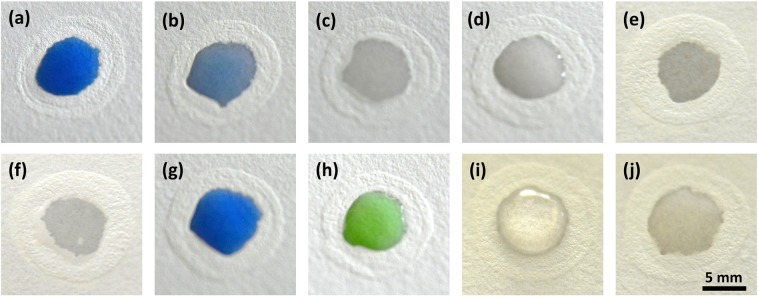
Chemical compatibility of barriers (**a**) Water (**b**) DMSO (**c**) Acetone (**d**) 40% Ethanol (**e**) DMF (**f**) Acetonitrile (**g**) PBS (**h**) 1N HCl (**i**) Glycerol (**j**) Tween- 20. Reagents for a, b, g, h were coloured with ink. Volume added in the device: 30 µl.

A wide gamut of research has already been reported on chemical compatibility of microfluidic devices to solvents and other reagents. Lee *et al*. have demonstrated the barrier compatibility of PDMS-based microfluidic devices with a comprehensive list of organic solvents^48^. Later, Dornelas *et al*. have proposed a simple rubber stamp based contact printing of PDMS on paper^49^. The authors have examined the barrier compatibility of PDMS and Wax with solvents and surfactants. Wang *et al*. used a different approach, where they have used sol-gel method and inkjet-printing to fabricate barriers on paper^50^. Furthermore, they compared chemical compatibility of Methylsilsesquioxane (MSQ) based hydrophobic barriers with wax and alkyl ketene dimer and concluded that MSQ barriers were not disrupted by surfactants. In order to use our method for bio-sensing or point-of-care testing, it is of paramount importance to study the fabricated barrier’s resistance or inertness to different reagents. For this, we used 10 devices per reagent and monitored the barrier disruption. Interestingly, the results revealed that 70% or higher devices have successfully confined the reagents without any disruption. Device’s compatibility with different chemicals is depicted in Fig. 3 and Supplementary Table 1. Through barrier compatibility test, we infer that our fabrication method holds great promise in confining most of the commonly used aqueous solutions and organic solvents.

### Surface Characterization

**Figure 4:**
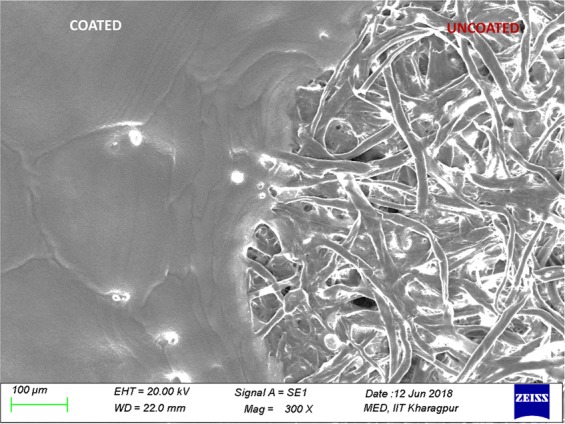
SEM image of Whatman^(R)^ cellulose filter paper (Grade 1); coated and uncoated with the liquid of correction pen.

Figure 4 shows the SEM micrographs of coated and uncoated part of the Whatman^(R)^ filter paper (Grade 1). The difference in the surface morphology corroborates to pore blockage by particles present in correction pen. To analyse the elemental composition of the coated and uncoated surface, we performed Energy Dispersive Spectroscopy and found that coated surface possess abundance of Titanium (Supplementary Fig. 2 and Table 2). To strengthen our statement, we have also carried out AFM studies on the barriers and plain paper surface (Supplementary Fig. 3). We have found that Root Mean Square height value of coated and uncoated is 0.145 µm and 0.758 µm respectively (data not shown). This result clearly attributes to the difference between the morphology of two surfaces. Overall, it is clear from the results that the components present in correction pen block the pores and mask the paper surface, which results in sample confinement.

**Figure 5:**
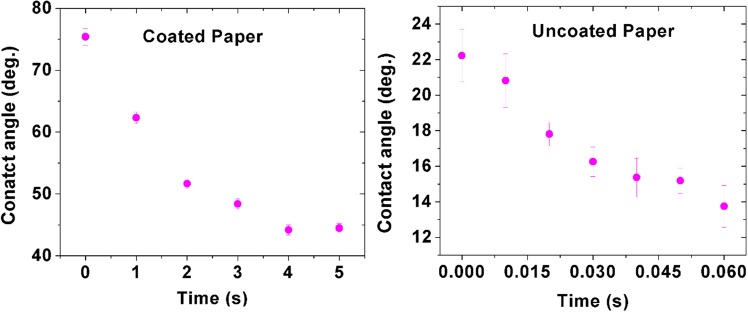
Water contact angle measurements of coated and uncoated paper surface. 3 samples were used for each data point. Average contact angle and ± Standard deviation were measured.

We have further studied the wetting property of coated and uncoated filter paper by water contact angle measurements. Figure 5 clearly indicates the difference in the surface wettability. The results reveal that the initial contact angle of coated and uncoated paper is 75° and 22° respectively. High contact angle of coated paper attributes to the hydrophobic nature of the surface (due to deposition of TiO_2_ particles), whereas the less contact angle of uncoated paper attributes to the hydrophilic nature of the surface. Time lapse images of contact angle measurements of both the surfaces are provided (Supplementary Fig. 4). Songok *et al*. have used liquid flame spray technique and coated titanium dioxide nanoparticles on paper. The authors reported that the coated TiO_2_ nanoparticles impart hydrophobicity on the paper surface. Moreover, the authors have observed variations in the wettability of paper surface by measuring the contact angle. They reported that uncoated paper surface is hydrophilic (CA~80°), whereas the TiO_2_ coated paper surface is super hydrophobic (CA~150°)^51^. The above finding is in good agreement with our proposed approach and supports the utility of simple titanium dioxide based correction pen for fabricating hydrophobic barriers.

**Figure 6:**
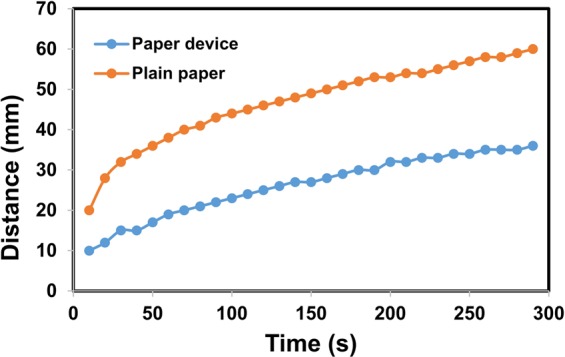
Comparison of water penetration rate between correction pen-based paper device and plain paper.

We also have characterized the surface by studying the water penetration rates of correction pen based hydrophilic channel and the plain filter paper. Interestingly, the water penetration profile in both fabricated device and the plain paper follow similar trends (Fig. 6). In our opinion, correction pen or Titanium-based composites, inarguably, can be added to the list of low-cost materials like PDMS and Wax.

### Biochemical assays

**Figure 7:**
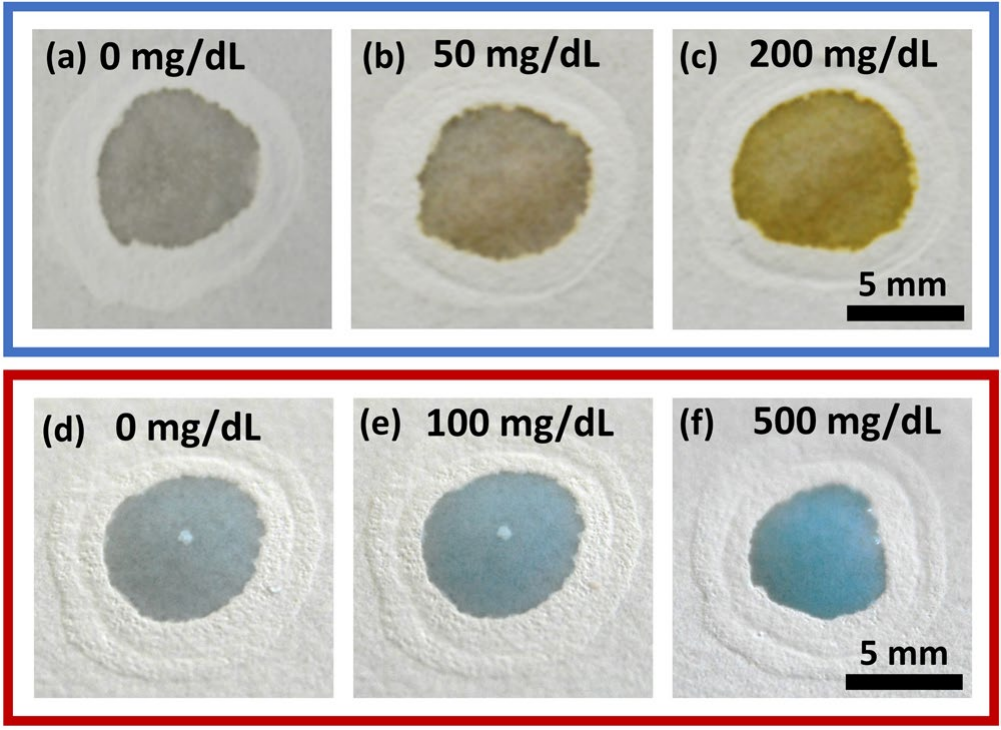
Standard colorimetric assays (**a**–**c**) Glucose (**d**–**f**) Protein.

To validate the functionality of the correction pen-based devices, we have performed basic colorimetric assays. Owing to the simplicity, we have chosen glucose and protein estimation using glucose oxidase enzymatic mixture and Bradford reagent respectively. We have studied the change in colour intensity by varying the glucose and protein concentration. For this, we have selected a wide range of concentration (0–300 mg/dl) and (0–2000 mg/dl) for standard glucose and protein respectively. Figure 7 suggests variation in colour intensity is proportional to the concentration of the analyte.

**Figure 8:**
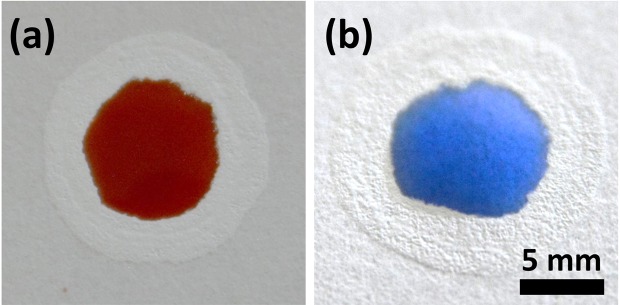
(**a**) Checking the functionality of the device by adding blood (**b**) Assessing the shelf-life of the device by adding 30 ul coloured water.

Moreover, upon addition of blood (20 µl) to the circular device, we observed no cross reactivity of blood sample with the barrier (Fig. 8a). This implies that the barrier’s composition limits surface interaction, confirming its suitability for any enzymatic or biochemical assays. Shelf-life of paper-based devices is always a crucial parameter for long-term storage in resource-limited settings. We observed no leakage or barrier disruption from the stored device (1 month) device (Fig. 8b). The main limitation of our fabrication method is the inability to confine alcohols and surfactants similar to wax barriers; however, this issue can be addressed^49^. Rajendra *et al*. have described wax barrier disruption due to surfactants and their importance in biological assays^52^. The authors developed silicone resins to circumvent such barrier disruption.

**Figure 9:**
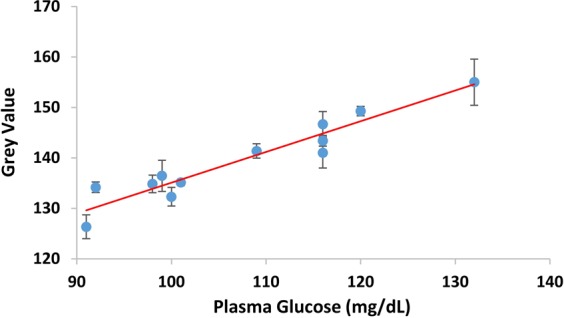
Relationship between grey value and different glucose concentrations in blood plasma.

Further, we have explored the applicability of the platform towards developing a quantitative assay for a very important blood parameter, namely, the blood glucose level. For this, we used blood samples of known glucose concentration (estimated through EM 360 automated clinical analyser). Later, we assessed the glucose levels in blood plasma and their relative colour intensities. 12 blood samples of varying glucose concentrations were collected. Each sample was deposited in 3 different devices and the average colour intensities ± standard deviations were measured. As it can be witnessed from Fig. 9, a linear increase in the grey scale value attributes to increase in the glucose concentration. All the reported data points agreed within 10% of the calibration curve (essentially, the straight line). During this study, no barrier leakage or disruption was observed. This suggests that our method can be easily employed for colorimetric detection of blood-based parameters through point-of-care testing.

## Conclusions

To summarize, in this study, we have described a quick, reliable and cost-effective one-step method to fabricate paper-based devices. We have used simple free-hand drawing to deposit Titanium dioxide based correction fluid and have blocked the pores of filter paper. The deposit exhibits unique resistance to different aqueous solutions and solvents. We have confirmed the workability or functionality of the device through standard glucose, protein and plasma glucose assays. The fabrication cost of one device is 0.0037 US $ and time is <10 s respectively. We are aware that our method has two limitations. The first one is minor toxicity. The second is inability to confine surfactants as well as alcohols. It is well known that the role of surfactants is restricted only to cell lysis and alcohols have negligible role in medical diagnostics. Henceforth, this method can safely be used as a simple substitute for paper-based analytical devices that are fabricated by other techniques, for common purposes. In addition, this may pave a way for developing frugal Titanium-based composites or inks for constructing paper-based device for bio-sensing applications compatible with the requirements of the economically deprived community at large.

## Methods and Materials

### Materials

Whatman^(R)^ cellulose filter paper (Grade 1) was purchased from GE Life sciences, India. Correction pens with tip size of 0.8 mm (Camlin, Faber Castle & Cello brands) and Fountain pen ink were obtained from local stationery. Glucose assay kit was supplied by Arkray Health Care, India. Protein Estimation kit was supplied by Himedia Laboratories, India. Triton-X was purchased from Loba Chemie. Ethanol, Isopropanol, Acetone, Acetonitrile, Dimethyl formamide, Sodium dodecyl sulphate were purchased from Merck, India. All other reagents and chemicals of analytical grade were purchased from Sigma-Aldrich, India.

### Fabrication of paper-based analytical devices

Correction pen was gently pressed in order to dispense fluid onto the filter paper (deposition on both the sides of the paper is advisable). The fabricated device was cured for 30 min at room temperature (25 °C). One side of the filter paper was sealed using 3 M adhesive tape. The thickness of plain and coated filter paper was measured using Mitutoyo Digital Micrometer (293–831).

### Image acquisition

Device images were captured using Nikon D5200 camera at a fixed distance. After acquisition, images were processed using FIJI and MS PowerPoint. Greyscale measurement for plasma glucose was performed using FIJI. Firstly, the images were converted to 8-bit grey scale. Secondly, the images were inverted to have a positive slope. Finally, mean grey value was measured.

### Surface characterization

SEM analysis was performed using Zeiss EVO 18, smart SEM software. Energy Dispersive Spectroscopy for Elemental analysis was carried out using EDAX (Ametek), TEAM software. Surface morphology of coated and uncoated filter paper was performed using an Agilent Technologies, AFM 5100 silicon nitride cantilever (PPP-NCL, Nanosensors Inc., USA) in tapping mode. Water penetration rate was assessed using a ruler, a camera and a timer. Water contact angle was recorded using Phantom V641 high-speed camera.

### Ethical approval and blood sample collection

An approval of ethical clearance (Approval No: IIT/SRIC/DR/2017 Dated: April 27, 2017) was obtained from Institute Ethical Committee (IEC) for experimenting with blood sample. The diabetic and normal blood samples were collected from B C Roy Technology Hospital, Indian Institute of Technology Kharagpur. An informed consent was received from all participants. Only one sample was collected from each participant after receiving the approval of informed consent from the patient or Guardians. All the experiments using human blood samples were performed according to the guidelines and regulations of Institute Ethical Committee (Indian Institute of Technology Kharagpur, India), Department of Biotechnology (Ministry of Science and Technology, Govt. of India) and Indian Council of Medical Research, India.

### Biochemical assays

Glucose assay was performed in this order. 10 µl of Glucose Oxidase enzyme mix was added to the fabricated device followed by the addition of 10 µl standard glucose of varying concentrations. For protein assay, 15 µl of Bradford reagent was added to the device. Later, 5 µl Bovine Serum Albumin of different concentrations were added. Conventional blood glucose level was measured using Erba Mannheim 360 Automated clinical chemistry analyser. Plasma was obtained by centrifuging at 3500 rpm for 5 min. In paper devices, 10 µl of Glucose Oxidase enzyme mix was added initially, followed by 10 µl of Plasma sample.

## Supplementary information

Supplementary experimental information
