# Supplementary material for: Fabricating Paper Based Devices Using Correction Pens
Source: Sci Rep. 2019 Feb 11;9:1752. doi: 10.1038/s41598-018-38308-6 (PMC6370803; doi:10.1038/s41598-018-38308-6)
Supplement: Supplementary file 1 — Supplementary experimental information [file 41598_2018_38308_MOESM1_ESM.pdf]

Supplementary Information

Fabricating Paper Based Devices Using Correction Pens

Naresh Kumar Mani[a,b,d‡], Anusha Prabhu[a‡], Sujay Kumar Biswas[c] and Suman Chakraborty[d,*]

[a] Department of Biotechnology, Manipal Institute of Technology, Manipal Academy of Higher Education, Manipal 576104, Karnataka, India
[b] Manipal-McGill Centre for Infectious Diseases, Manipal Academy of Higher Education, Manipal 576104, Karnataka, India
[c]School of Medical Science and Technology, Indian Institute of Technology Kharagpur 721302, India
[d]Department of Mechanical Engineering, Indian Institute of Technology Kharagpur 721302, India

Barrier's compatibility

Compatibility of the fabricated device was assessed using different chemicals. For each reagent, ten circular devices were used to check the barrier's intactness. The device was observed for 15 min or until evaporation. Water, DMSO, PBS, 1N HCl were coloured with ink.

Supplementary Table 1. Barrier resistance study and the list of reagents used.

Reagent	Volume	Barrier intactness
Water, DMSO, Acetone,40% Ethanol, DMF, Acetonitrile, Phosphate Buffer Saline, 1NHCl, Glycerol, &Tween 20	30 µl	Yes
1% SDS, 1% Triton-X	10-30 µl	Disrupted
2-Propanol, Ethanol, Methanol	10-30 µl	Disrupted

Supplementary Table 2. Elemental composition using EDS

Substrate	Element	Weight %	Atomic %
**Coated**	C	28.90	44.62
	O	35.08	40.65
	**Al**	**1.73**	**1.19**
	**Si**	**1.00**	**0.66**
	**Ti**	**33.29**	**12.89**
**Uncoated**	C	49.60	56.73
	O	50.40	43.27

Wicking of ink in fabricated device

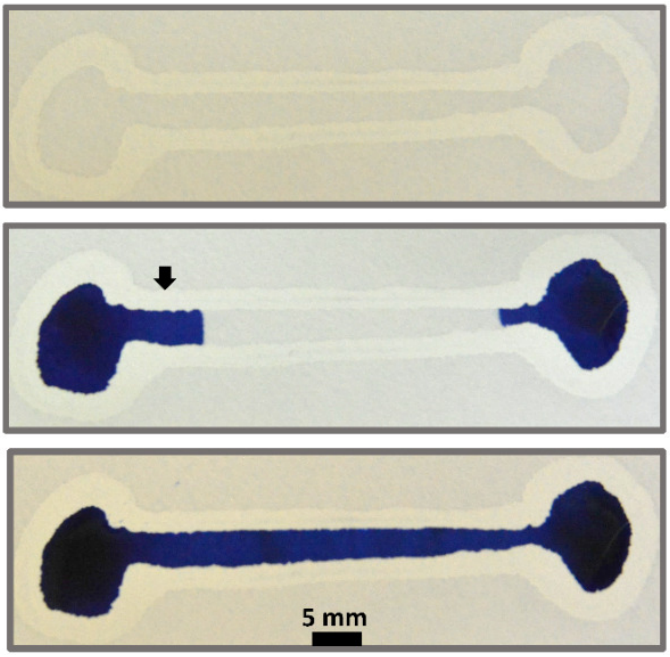

Supplementary Figure 1: Wicking of water and ink in dumb-bell shape channel.

EDS analysis

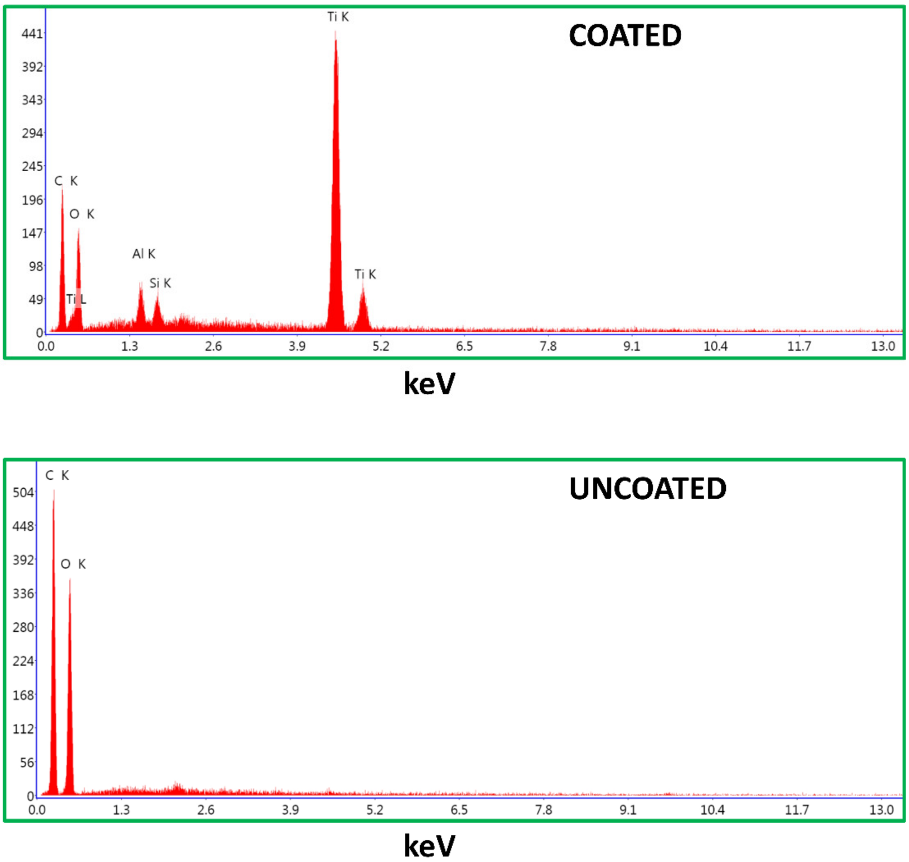

Supplementary Figure 2: EDS analysis of Coated and Uncoated Whatman[(R)] cellulose filter paper (Grade 1).

AFM analysis

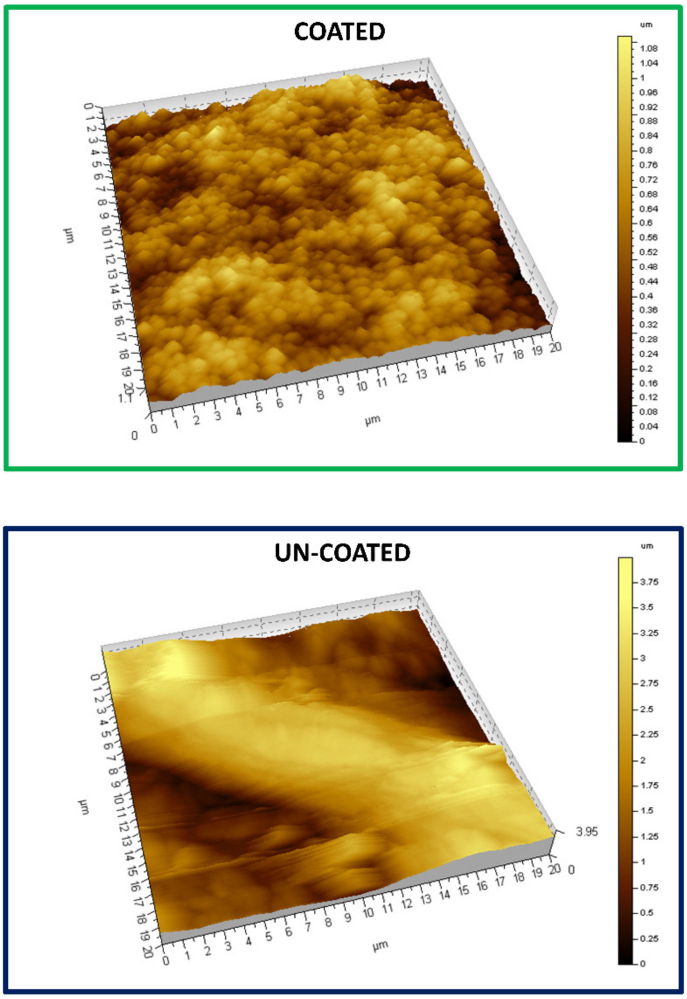

Supplementary Figure 3: AFM analysis of Coated and Uncoated Whatman(R) cellulose filter paper (Grade 1).

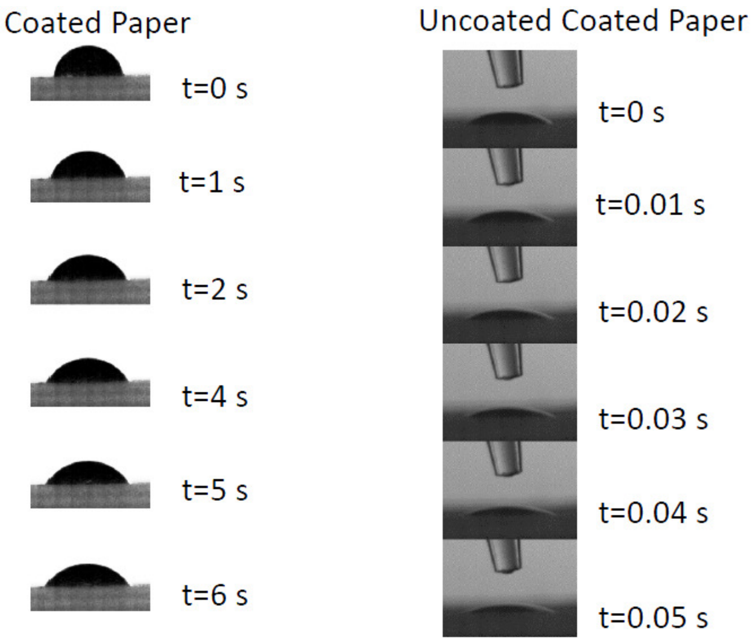

Supplementary Figure 4: Water Contact Angle measurements of Coated and Uncoated Whatman$^{(R)}$ filter paper (Grade 1).
